# Epidemiology and long-term survival of pulmonary arterial hypertension in the Czech Republic: a retrospective analysis of a nationwide registry

**DOI:** 10.1186/1471-2466-14-45

**Published:** 2014-03-15

**Authors:** Pavel Jansa, Jiri Jarkovsky, Hikmet Al-Hiti, Jana Popelova, David Ambroz, Tomas Zatocil, Regina Votavova, Pavel Polacek, Jana Maresova, Michael Aschermann, Petr Brabec, Ladislav Dusek, Ales Linhart

**Affiliations:** 1 1st Faculty of Medicine, 2nd Medical Department, Clinical Department of Cardiology and Angiology, Charles University, Prague, Czech Republic; 2 Institute of Biostatistics and Analyses at the Faculty of Medicine and the Faculty of Science, Masaryk University, Brno, Czech Republic; 3 Department of Cardiology, Institute of Clinical and Experimental Medicine-IKEM, Prague, Czech Republic; 4 Department of Cardiac Surgery, Na Homolce Hospital, Prague, Czech Republic; 5 Department of Cardiology, University Hospital, Brno Bohunice, Brno, Czech Republic

**Keywords:** Pulmonary arterial hypertension, Epidemiology, Survival, Czech Republic, National registry

## Abstract

**Background:**

Pulmonary arterial hypertension (PAH) is a severe and progressive disease characterized by increased pulmonary vascular resistance, ultimately leading to right heart failure and death. Epidemiological data from national registries are growing worldwide, but are still unavailable in Eastern Europe.

**Methods:**

A PAH registry was initiated in January 2007 using a nationwide network of echocardiographic centers and four diagnostic centers that specialize in PAH. All patients aged above 18 years, diagnosed with PAH and monitored between January 2000 and December 2007 were included. Patients diagnosed with PAH between January and December 2007 were classified as incident. The survival analyses were performed up to the end of 2010. Prognostic factors at the time of diagnosis were identified using uni- and multivariable Cox proportional hazard models.

**Results:**

Overall, 191 patients were included (100 prevalent cases, 91 incident cases). Patients were predominantly female (n = 125) and had a mean age of 51.9 ± 16.9 years. Incident patients were significantly older at the time of diagnosis than prevalent patients (p < 0.001). Most patients (60.7%) had idiopathic PAH; 20.4% had PAH associated with congenital heart disease and 11.4% had PAH associated with connective tissue disease. Estimates of prevalence and incidence of PAH in adults were 22.4 cases per million and 10.7 cases per million per year, respectively. The 1-, 2- and 3-year survival rates in the incident PAH cohort were 89% (95% confidence intervals [CI] 83–95%), 78% (95% CI 70–87%) and 74% (95% CI 65–83%), respectively. Lower survival rates were significantly associated with higher age (hazard ratio [HR] 6.6 95% CI 1.4–30.9) and lower creatinine clearance (HR 3.3 95% CI 1.1–9.7).

**Conclusion:**

This is the first study in Eastern Europe to describe the prevalence, incidence and survival of patients with PAH from a national representative registry. This registry from the Czech Republic highlights that diagnosis of PAH is frequently made late in the disease continuum when patients have significant functional impairment.

## Background

Pulmonary arterial hypertension (PAH) is a chronic, progressive and potentially fatal disease of the pulmonary vasculature that leads to heart failure. PAH is a clinical condition characterized by the presence of pre-capillary pulmonary hypertension. It is defined as an increase in mean pulmonary arterial pressure (PAP) ≥25 mmHg at rest in the absence of significantly elevated pulmonary capillary wedge pressure (PCWP ≤15 mmHg) as assessed by right heart catheterization (RHC) [1]. PAH is classified into a number of categories according to etiology [1]. These are: idiopathic PAH, heritable, induced by exposure to drugs and toxins, or associated with other conditions such as connective tissue disease (APAH-CTD) or congenital heart disease (APAH-CHD). At the recent World Symposium on Pulmonary Hypertension (PH [Nice 2013]) there were some suggestions to update the classification of PH, for example, to classify patients with hemolytic anemia into Group 5 [2]; in this paper we have classified patients according to the current guidelines.

Population-based estimates of PAH incidence and prevalence are not available. However, as in other rare diseases, patient registries provide valuable epidemiological information about PAH [3]. Data from PAH registries suggest estimates for PAH and idiopathic PAH incidence of 2–8 and 1–2 cases per million population per year, respectively [4-10]. The Orphanet 2012 report estimates the prevalence of idiopathic PAH in Europe to be about 6 per million population [11]. Based on information collected in PAH registries, prevalence estimates of PAH and idiopathic PAH range between 15 to 26 cases per million adults and 6 to 9 cases per million adults, respectively [4-10].

The data from the US National Institutes of Health (NIH) registry over 20 years ago provided the first estimates of survival rates in PAH and showed that the median survival rate was 2.8 years after diagnosis [12]. Several treatment options have become available for management of PAH since the NIH registry was published. In the Czech Republic epoprostenol became available in 2000, followed by treprostinil, iloprost and bosentan in 2003, sildenafil in 2006 and ambrisentan became available in 2011. Until 2006 the use of PAH-specific drugs in the Czech Republic was restricted to a limited number of patients. Since 2006 PAH-specific therapy is prescribed in specialist centers in the Czech Republic with no restriction on the number of patients who can receive therapy. However, despite clear improvements in patient outcomes, the disease remains incurable [5].

To date, national registries for PAH are limited to the USA, Western Europe, China and Australia [3]. The only available epidemiology data about PAH in the Czech Republic have been from a retrospective analysis of the number of patients diagnosed in cardiology and pneumology clinics during the period 1980–1999 [13]. Since then an effective network of echocardiographic and PAH expert centers has been developed. The objectives of the current study were to estimate the prevalence and incidence of PAH in the Czech Republic, to describe the clinical characteristics of patients with PAH included in the national registry during the modern treatment era and to estimate survival in a cohort of incident patients. Prognostic factors for survival were also explored in the cohort of incident PAH patients.

This study estimates the prevalence, incidence and long-term survival of PAH patients in the Czech Republic.

## Methods

### Study design

A system of centralized care in four expert centers, which share a unique data collection system, was set up in 2000 in the Czech Republic. In 2007, this system was extended to improve the detection of PAH by establishing a network of referring echocardiographic laboratories. Currently all patients in the Czech Republic with PAH, as recognized during echocardiographic assessment, have the final diagnosis confirmed by RHC in one of the two specialized centers (Charles University, 1st Faculty of Medicine, 2nd Medical Department, Clinical Department of Cardiology and Angiology, Prague; Cardiology Center, Institute of Clinical and Experimental Medicine-IKEM, Department of Cardiology, Prague, Czech Republic). Long-term follow-up is then provided at either of the two specialized centers or at two other expert centers (Na Homolce Hospital, Department of Cardiac Surgery, Prague; University Hospital, Brno Bohunice, Department of Cardiology, Brno).

In this retrospective analysis, all consecutive patients aged ≥18 years with a diagnosis of Group 1 PAH made either between January and December 2007 (incident cases) or before January 2007 (prevalent cases) were included. Of the patients diagnosed before 2007, only those who survived to January 2007 were included in the analysis. Patients diagnosed after 2007 were not included in this study as access to the database information for all centers diagnosing PAH was not available after 2007. Patients were classified as having either idiopathic PAH, heritable PAH, APAH-CTD, APAH-CHD or ‘other’ (‘other’ patients included patients with portal hypertension or PAH associated with hemolytic anemia) according to the 2009 international clinical classification of pulmonary hypertension [1]. PAH was defined as mean PAP ≥25 mmHg at rest and PCWP ≤15 mmHg.

All patients meeting the definition for PAH were eligible for the analysis. Time of diagnosis was defined as the date on which diagnosis by RHC was made. To be eligible for the analysis, RHC must have been performed before study entry. Patients were ineligible if they were aged under 18 years at the time of diagnosis. In order to assess a homogenous population, patients with abnormal ventilation parameters (forced vital capacity, total lung capacity or forced expiratory volume in 1 second <60% of normal values [14]) and patients with significant left heart disease (recognized as left ventricular ejection fraction <50% and significant left-sided valvular disease) were excluded from the analysis. Patients with chronic thromboembolic pulmonary hypertension identified using ventilation-perfusion scintigraphy were also excluded.

An acute vasodilator challenge was performed during RHC to assess vasoreactivity. This was assessed by administration of an intravenous synthetic prostacyclin analog. A positive test was defined as a drop in mean PAP greater than 10 mmHg leading to a value <40 mmHg without a decrease in cardiac output.

Clinical, hemodynamic and pulmonary parameters were recorded at the time of diagnosis. A functional assessment of 6-minute walk distance (6MWD) and New York Heart Association (NYHA) functional class was also conducted. The time between start of symptoms (information provided by the patient) and diagnosis was assessed.

### Analysis

Standard descriptive statistics were applied in the analysis. Mean ± standard deviation (SD) or median (interquartile and 5th–95th percentile ranges) were used to describe continuous parameters. Comparisons between groups of patients were performed using Mann–Whitney U test for continuous parameters. Categorical variables were described by frequencies, and comparisons between groups were performed using maximal likelihood chi-square test of Fisher’s exact test. The limit of statistical significance was set as p = 0.05.

Newly diagnosed patients (January 1 to December 31, 2007) were included for calculation of the incidence, while all living patients by January 1, 2007 were used to estimate prevalence. Rates were standardized per one million adult persons using the National Czech census data as a reference [15] with a 95% confidence interval reported based on a Poisson distribution. The denominator of the population used to calculate incidence was 8,522,012 (the adult Czech population in 2007).

For the survival analysis, an endpoint of all-cause mortality was used and patients were followed to their last visit in 2010. Survival was estimated from time of diagnostic RHC to 6, 12, 18, 24 and 36 months post-diagnosis using the Kaplan-Meier method and survival curves compared with the log-rank test [16].

The relationship between potential prognostic variables measured at the time of diagnosis and mortality was assessed in the incidence cohort of patients with idiopathic or heritable PAH using uni- and multivariable Cox proportional hazard models. The selection of variables for the multivariable model was based on univariate statistical significance <0.1 followed by redundancy analysis using correlation analysis of predictors and a forward stepwise algorithm.

### Ethics committee approval

The study was discussed with the Ethics Committee of the General University Hospital in Prague. As it is a retrospective analysis of anonymized data from routine clinical practice, Czech legislation does not require approval from an ethics committee.

## Results

### Patient population

Between January 2000 and December 2007, 191 patients were diagnosed with PAH and included in the registry (79.6% at the 2nd Department of Internal Medicine, General University Hospital and 1st Medical Faculty of Charles University, Prague and 20.4% at the Cardiology Center, Institute for Clinical and Experimental Medicine, Prague). Incident patients accounted for 47.7% (n = 91) of all patients.

Overall, 65% (n = 125) of diagnosed patients were female and the mean age of the population was 51.9 ± 16.9 years. The majority of patients were diagnosed between the ages of 51 and 70 years. The incident group was significantly older at the time of diagnosis than the prevalent group (p < 0.001). Baseline clinical, hemodynamic and pulmonary parameters in the prevalent and incident groups are shown in Table 1.

**Table 1:** Clinical, hemodynamic and pulmonary parameters in the prevalent and incident populations

	**Prevalent cases n = 100**	**Incident cases n = 91**	**p**^**†**^
**Female sex, n (%)**	65 (65.0)	60 (65.9)	0.892
**NYHA FC**			0.229^‡^
**I + II, n (%)**	34 (34.0)	21 (23.1)	
**III, n (%)**	64 (64.0)	67 (73.6)	
**IV, n (%)**	2 (2.0)	3 (3.3)	
Age^*^ (years)	47.0 (17.0) 47.8 (34.5–60.7/19.4–73.8)	57.3 (15.1)^†^ 59.8 (48.9–68.8/27.4–75.7)	<0.001
BMI^*^ (kg/m^2^)	26.2 (5.4) 26.0 (21.8–29.7/18.3–35.9)	27.8 (6.3) 26.5 (23.2–31.9/19.5–37.9)	0.100
6MWD^*^ (m)	332.4 (123.0) 356.0 (246.0–420.0/150.0–511.0)	314.0 (117.0) 332.5 (239.0–403.0/128.0–474.0)	0.313
RAP^*^ (mmHg)	10.0 (5.3) 9.0 (7.0–12.0/3.0–20.0)	9.8 (5.2) 9.0 (6.0–12.0/2.0–21.0)	0.934
mPAP^*^ (mmHg)	65.8 (84.8) 56.5 (46.0–66.0/32.0–89.0)	51.8 (19.7)^†^ 47.0 (37.0–64.0/27.0–91.0)	0.004
PASP^*^ (mmHg)	88.2 (24.6) 91.0 (70.0–103.0/45.0–134.0)	80.7 (28.5)^†^ 77.0 (58.0–97.0/40.0–132.0)	0.017
PCWP^*^ (mmHg)	10.5 (3.2) 11.0 (8.0–13.0/5.0–15.0)	11.9 (3.1)^†^ 12.0 (10.0–15.0/7.0–15.0)	0.003
Cardiac index^*^ (l/min/m^2^)	1.9 (0.7) 1.7 (1.3–2.4/0.8–3.3)	2.4 (0.8)^†^ 2.4 (1.9–2.9/1.1–3.9)	<0.001
CO^*^ (l/min)	3.4 (1.5) 3.2 (2.3–4.3/1.6–6.4)	4.3 (1.6)^†^ 3.9 (3.2–5.4/2.1–7.1)	<0.001
**SvO**_2_^*^ (%)	92.5 (5.5) 94.5 (90.2–96.4/78.0–98.1)	91.7 (6.8) 93.5 (89.9–96.2/79.0–98.0)	0.461
**SvO**_2_^*^ (%)	66.4 (7.5) 66.0 (61.0–72.0/55.8–78.7)	70.0 (8.6)^†^ 72.0 (64.6–76.0/56.0–81.2)	0.002
LVEF^*^ (%)	60.9 (8.4) 61.5 (55.0–67.0/46.0–74.0)	59.3 (7.5) 58.0 (55.0–64.0/45.0–72.0)	0.199
PAR^*^ (WU)	20.1 (31.1) 16.0 (9.3–21.4/4.2–42.2)	10.8 (7.1)^†^ 8.5 (5.6–15.2/3.0–25.8)	<0.001
S-creatinine^*^ (μmol/l)	95.3 (43.1) 88.7 (71.6–99.0/59.1–173.5)	97.2 (49.8) 81.2 (71.0–104.0/56.0–193.0)	0.895
Creatinine clearance^*^ (ml/min)	90 (30) 84 (66–108/42–144)	78 (36) 78 (60–96/30–138)	0.186

NYHA, New York Heart Association; BMI, body mass index; 6MWD, 6-minute walk distance; RAP, resting arterial pressure; mPAP, mean pulmonary arterial pressure; PASP, pulmonary artery systolic pressure; PCWP, pulmonary capillary wedge pressure; SaO_2_,oxygen saturation of arterial blood; SvO_2_, oxygen saturation of central venous blood; LVEF, left ventricular ejection fraction; PAR, pulmonary arteriolar resistance; CO, cardiac output.

^*^Mean (SD), median (25^th^–75^th^/5^th^–95^th^ percentile).

^†^Mann–Whitney U test for continuous parameters; maximal likelihood chi-square test or Fisher’s exact test for categorical parameters. ^‡^P-value represents significance of NYHA FC between incident and prevalent cases.

The most frequently diagnosed type of PAH was idiopathic (60.7%) followed by APAH-CHD (20.4%) and then APAH-CTD (11.0%) (Table 2). Of the PAH-CHD patients, 21 (53.8%) had Eisenmenger syndrome, four had PAH with left-to-right uncorrectable shunts and 14 had post-operative PAH. The patients had various defects: atrial septal defects (n = 11), ventricular septal defect (n = 17), patent ductus arteriosus (n = 6) and others (atrioventricular septal defect, truncus arteriosus or aortopulmonary window [n = 5]). There was no significant difference in the proportion of patients with each etiology in the prevalent and incident groups. The acute pulmonary vasoreactivity test, conducted in all patients was positive in 3.1% of patients (n = 6): five cases with idiopathic or heritable PAH and one case with APAH-CTD. Vasoactive potential was not observed in the other PAH etiologies.

**Table 2:** Proportion of PAH subtypes at time of diagnosis

**PAH subtype**	**All cases n = 191**	**Prevalent cases n = 100**	**Incident cases n = 91**	p^*^
Idiopathic, n (%)	116 (60.7)	67 (67.0)	49 (53.8)	0.177
Heritable, n (%)	7 (3.7)	3 (3.0)	4 (4.4)	
APAH-CHD, n (%)	39 (20.4)	16 (16.0)	23 (25.3)	
APAH-CTD, n (%)	21 (11.0)	12 (12.0)	9 (9.9)	
Other^†^, n (%)	8 (4.2)	2 (2.0)	6 (6.6)	

APAH-CHD, pulmonary arterial hypertension associated with congenital heart disease; APAH-CTD, pulmonary arterial hypertension associated with connective tissue disease.

^†^Other PAH etiologies included: portal hypertension and PAH associated with hemolytic anemia.

^*^Difference between prevalent and incident cases maximal likelihood chi-square test.

Most of the patients (71.2%) were in NYHA functional class III or IV at the time of diagnosis. This is reflected in the patients’ treatments – nearly all patients were receiving treatment with PAH-specific therapies, and the most common treatment was sildenafil, followed by bosentan (Table 3). There was little difference between the treatments in patients who were incident compared with prevalent patients.

**Table 3:** Treatment of incident and prevalent patients following diagnosis

**Treatment, n**	**Incident (n = 91)**	**Prevalent (n = 100)**
Calcium channel blockers	3	2
Bosentan	27	25
Sildenafil	35	31
Treprostinil	4	5
Epoprostenol	-	1
Combination therapy	11	26
Bosentan + sildenafil	3	14
Bosentan + iloprost	2	-
Sildenafil + iloprost	2	2
Sildenafil + treprostinil	2	4
Sildenafil + epoprostenol	2	4
Epoprostenol + sildenafil + bosentan	-	2
Investigational drugs	7	5
No specific therapy	4	5

In the 156 patients with available data on the time of symptom onset, the mean time from the manifestation of symptoms until a confirmed diagnosis was 38.7 ± 47.7 months across the entire group, 36.4 ± 42.3 months in idiopathic PAH patients, 52.0 ± 62.8 months in APAH-CHD patients, 30.8 ± 45.8 months in APAH-CTD patients and 50.8 ± 92.9 months in heritable PAH patients. The difference in time from the manifestation of symptoms between the prevalent and incident cases was not statistically significant (Table 4). Median follow up time after inclusion in the registry was 38 months.

**Table 4:** Time from appearance of first PAH symptoms and diagnosis

**PAH subtype**	**Time between first symptoms and diagnosis (months)**			
	**Total (n = 156)**	**Prevalent cases (n = 79)**	**Incident cases (n = 77)**	p^*^
**Idiopathic**				
Mean (SD)	36 (42)	36 (42)	37 (44)	0.742
Median (25^th^-75^th^; 5^th^-95^th^)	23 (10–49/2–120)	22 (7–48/1–120)	24 (10–60/2–96)	
**APAH-CHD**				
Mean (SD)	52 (63)	31 (45)	62 (69)	0.244
Median (25^th^-75^th^; 5^th^-95^th^)	24 (8–96/0–251)	21 (2–24/0–120)	36 (24–96/1–251)	
**APAH-CTD**				
Mean (SD)	31 (46)	47 (59)	13 (8)	0.139
Median (25^th^-75^th^; 5^th^-95^th^)	12 (8–24/2–192)	24 (12–60/6–192)	12 (6–15/2–24)	
**Heritable**				
Mean (SD)	51 (93)	15 (13)	69 (114)	0.605
Median (25^th^-75^th^; 5^th^-95^th^)	13 (10–24/5–240)	15 (5–24/5–24)	13 (10–14/10–240)	
**Total**				
Mean (SD)	39 (48)	36 (43)	42 (52)	0.523
Median (25^th^-75^th^; 5^th^-95^th^)	24 (10–49/1–120)	22 (8–48/1–120)	24 (10–60/2–120)	

APAH-CHD, pulmonary arterial hypertension associated with congenital heart disease; APAH-CTD, pulmonary arterial hypertension associated with connective tissue disease.

^*^Difference between prevalent and incident cases Mann–Whitney U test.

### Incidence and prevalence

The estimated incidence of PAH in adults in the Czech Republic was 10.7 cases per million persons overall in 2007 (7.5 per million men and 13.7 per million women). The incidence of idiopathic/heritable PAH was 6.2 per million persons (6.1 per million men and 7.3 per million women). The estimated prevalence of PAH in the Czech adult population was 22.4 cases per million persons overall (16 per million men and 28.5 per million women). The estimated prevalence of idiopathic/heritable PAH was 14.4 per million persons overall (11.1 per million men and 17.5 per million women).

### Survival of incident PAH patients

**Figure 1:**
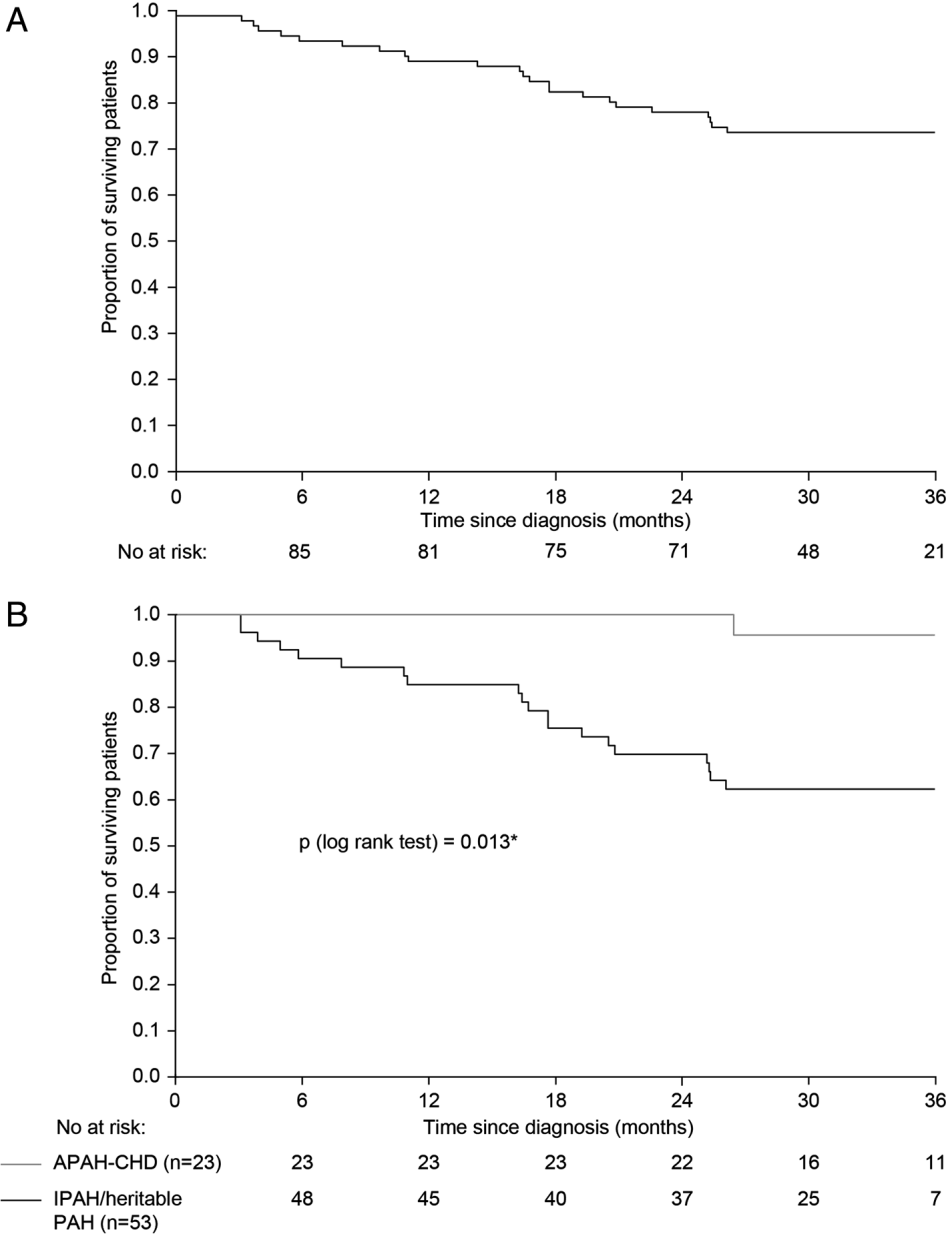
**Overall survival in the incident population. A)** All patients with PAH; **B)** Patients with idiopathic or heritable PAH (n = 53) or APAH-CHD (n = 23).

In the incident population, the 1-, 2- and 3-year survival rates were 89%, 78% and 74%, respectively (Figure 1A). When the subgroup of patients with idiopathic/heritable PAH was analyzed, the 1-, 2- and 3-year survival rates were 85%, 70% and 62%, respectively (Figure 1B). For the APAH-CHD subgroup, survival rates were 100%, 100% and 96%, respectively (Figure 1B).

**Figure 2:**
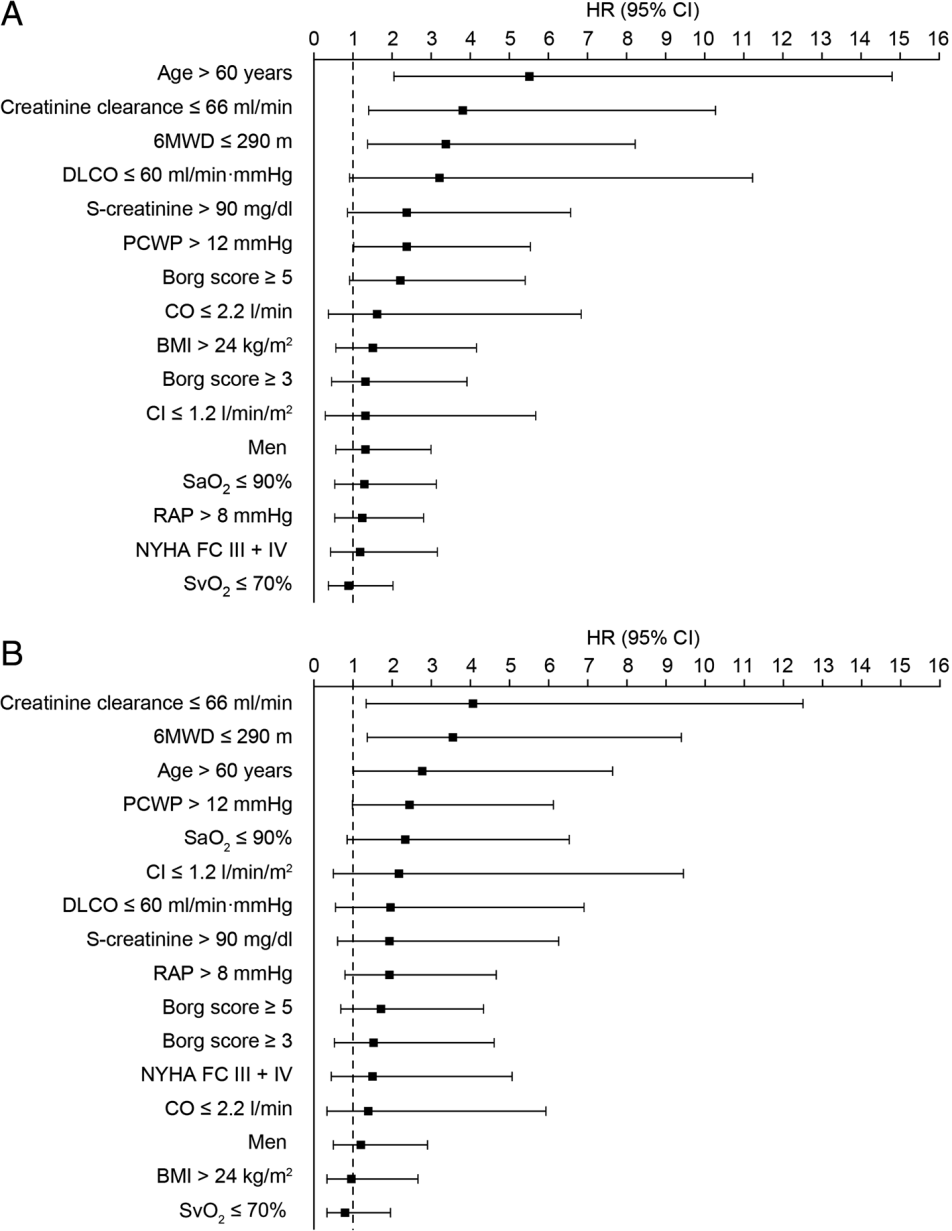
**Factors associated with survival in the incident population. A)** All patients (n = 91); **B)** Patients with idiopathic or heritable PAH (n = 53). Footnote: Optimal cut-off levels for parameters included in the Cox proportional hazard methods were identified using time-dependent receiver operating characteristic analysis [16]. The selection of variables for the multivariate model was based on univariate statistical significance <0.1 followed by redundancy analysis and a forward stepwise algorithm.

The univariate analysis of the incident group of PAH patients revealed significantly shorter survival rates in patients >60 years of age (hazard ratio and 95% confidence intervals [HR 95% CI] 5.5 [2.1, 14.8], p < 0.001), 6MWD ≤290 m (HR 3.4 [1.4, 8.2], p = 0.007), PCWP >12 mmHg (HR 2.4 [1.0, 5.5], p = 0.046) or with creatinine clearance ≤66 ml/min (HR 3.8 [1.4, 10.3], p = 0.008) (Figure 2A). The majority of these factors were also associated with shorter survival rates in a subgroup of incident patients with idiopathic/heritable PAH; the only exception being age (HR [95% CI] 2.8 [1.0, 7.6], p = 0.049) (Figure 2B). The other factors investigated had no significant influence on survival. The multivariate analysis of the incident group of PAH patients revealed significantly shorter survival rates in patients >60 years of age (HR 6.6 (1.4; 30.9), p = 0.016) or with creatinine clearance ≤66 ml/min (HR 3.3 (1.1; 9.7), p = 0.027). It was generally observed that in patients with idiopathic/heritable PAH and S-creatinine levels ≤90 mg/dl, that right atrial pressure was significantly lower when compared with S-creatinine >90 mg/dl (mean 5.8/median 5 vs mean 10.5/median 8, p = 0.013 – Mann–Whitney U test).

## Discussion

Our registry data estimated the annual incidence of PAH in the Czech adult population as 10.7 per million persons and the prevalence as 22.4 per million persons. The incidence rate is higher than the range of 2–8 per million reported by other registries while the prevalence is in the range previously reported [4-10]. The higher number of newly diagnosed incident cases in 2007 was confirmed by high diagnosis rates in the following years: The General University Hospital, Prague diagnosed 70 patients in 2008 and 63 patients in 2009 (P Jansa, personal communication, 2014). The number of newly diagnosed patients in 2007 (and in 2008 and 2009) compared with 2000–2006, could be explained by a higher referral rate after 2006, due to the wider availability of PAH-specific drugs after 2006. This high number of incident cases might also be explained by an efficient system for referring patients with suspected PAH, in a country with a relatively small population. The annual incidence of idiopathic and heritable PAH was 6.2 per million persons and the prevalence was 14.4 per million. Our results highlight that PAH remains a rare disease in Eastern Europe.

### Patient characteristics of the Czech population

Idiopathic/heritable PAH accounted for the majority of diagnoses (64%), while rates of APAH-CHD and APAH-CTD were lower and similar. In the French registry (n = 674), for comparison, 39% of patients had idiopathic PAH, 4% had familial (heritable) PAH, 15% had APAH-CTD, 11% had APAH-CHD, 9.5% had PAH associated with anorexigen use, 9.5% had portal hypertension associated PAH and 6.2% had human immunodeficiency virus-associated PAH [5]. In the Czech cohort of incident cases, APAH-CHD accounted for a greater proportion of PAH diagnoses than seen in the prevalent population. One explanation for the increased diagnosis rate of APAH-CHD is that effective PAH therapies were available in 2007 for this subgroup that had not been available at the earlier part of the study. It is possible that clinicians were more likely to refer patients for a PAH investigation once suitable therapy had become available.

As seen in previous registries, almost two-thirds of PAH patients were female with the mean age of diagnosis in the Czech population being approximately 52 years. The mean age was 10 years higher in the incident population than in the prevalent population, which seems to be a characteristic of the patient populations included in several contemporary registries [8]. This is unlikely to result from differences in disease course, but more likely to be a consequence of increased awareness of PAH and the availability of effective PAH therapies which could result in the consideration of a PAH diagnosis. Overall, patients had experienced PAH symptoms for over 3 years (mean 38 months) before a diagnosis was made, although this varied depending on PAH etiology. The trend towards shortening the time between the first sign of symptoms and diagnosis of APAH-CTD incident patients may result from the introduction of a systematic PAH screening program in patients with systemic sclerosis in 2007 and who are, therefore, at risk of APAH-CTD. The longer duration before diagnosis in patients with heritable PAH or APAH-CHD may be explained by their potentially long history of relevant symptoms with many experiencing fatigue or dyspnea since childhood and some patients never being completely asymptomatic; however this does have to be balanced with the increased suspicion of PAH in these patient groups.

### Hemodynamic characteristics of the Czech population

The hemodynamic parameters in Czech patients were characterized by severe precapillary pulmonary hypertension with low cardiac output and high pulmonary arteriolar resistance. Similar to other registries, the incidence of patients with positive vasoreactivity was low and might be related to late diagnosis and advanced disease. The low incidence of patients with positive vasoreactivity may also be related to the higher proportion of patients with PAH-CHD in the Czech registry (20.4%); data from the French registry have previously shown that patients with PAH-CHD have a lower probability of vasoreactivity versus patients with idiopathic PAH [5].

Incident cases on average were classified with more severe NYHA FC (although this was not significant) and patients were generally older than prevalent cases. However, their hemodynamics were typically better or comparable to the prevalent cases. A possible explanation for this is that the prevalent patients, despite poorer hemodynamics, have a better NYHA FC, as they are survivors and might be better adapted to respond, or are better responders to PAH-specific therapies. French registry data also found prevalent cases to typically have higher mPAP, but lower FC compared with incident cases [5].

### Survival of PAH patients in the Czech Republic

To date, this is the first representative study in the Eastern Europe that describes the survival rates of patient with PAH. Survival of PAH patients has been estimated in other national registries in the USA, Western Europe, China and Australia, but caution must be taken when comparing epidemiological data from different countries as the methodology can be variable. In the Registry to Evaluate Early and Long-term PAH Disease Management (REVEAL), 1-year survival in the combined incident/prevalent population of patients with idiopathic or heritable PAH was 91.0% (95% CI 89.9–92.1%) [17]. In the incident cohort of patients with idiopathic, heritable or anorexigen-associated PAH in the French registry, the survival estimate at 1 year was 86% and at 3 years was 55% [18]. In a registry of incident patients in UK and Ireland, the 1 and 3-year survival rates were 93% and 73%, respectively [9]. In the Czech analysis, lower survival in the overall PAH patient cohort was associated with higher age and lower creatinine clearance. The analysis showing that the patients with lower creatinine levels had significantly lower right atrial pressure versus patients with higher creatinine levels might, in part, explain why this was associated with survival. Patients with APAH-CHD also had the best prognosis when compared with patients with idiopathic or heritable PAH. These findings confirm previous analyses and the importance of PAH etiology on patient outcomes [19-21].

The limitations of the retrospective data from our observational study should be acknowledged. These include the potential loss of patients to follow-up and the difficulty defining the time of diagnosis in prevalent patients. It is also important to note that only expert centers were included in the registry and, therefore, some patients in the early stages of PAH may have been excluded. However, the well-defined process for PAH diagnosis in the Czech Republic is a definite strength of the study, as it allowed us to study the epidemiology of PAH during a period when considerable advances in PAH diagnosis were occurring. It should also be noted that due to the advances in diagnosis during this period and the increase in incident cases since 2007, the prevalence of PAH in the Czech Republic will need to be reevaluated in the near future.

## Conclusion

This is the first epidemiological study of PAH in the Czech Republic in the modern treatment era, increasing the knowledge on the epidemiology of PAH worldwide. It confirms that diagnosis of PAH is frequently made late in the disease continuum so that patients have significant functional impairment. Survival rates were similar to those seen in previous studies and shorter survival rates were observed in patients over the age of 60 years at the time of diagnosis with low creatinine levels. As the registry is ongoing it will continue to provide clinicians with data that will be valuable when managing PAH patients in the Czech Republic.

## Abbreviations

APAH-CHD: Pulmonary arterial hypertension associated with congenital heart disease; APAH-CTD: Pulmonary arterial hypertension associated with connective tissue disease; CI: Confidence interval; HR: Hazard ratio; mPAP: Mean pulmonary arterial pressure; NYHA: New York Heart Association; PAH: Pulmonary arterial hypertension; PCWP: Pulmonary capillary wedge pressure; RHC: Right heart catheterization; SD: Standard deviation; 6MWD: 6-minute walk distance.

## Competing interests

PJ has received honoraria, consultancy fees, and grants from Actelion Pharmaceuticals Ltd, Pfizer, Bayer, United Therapeutics, and AOP Orphan Pharmaceuticals; HA-H has been an investigator of clinical studies sponsored by Actelion Pharmaceuticals Ltd, Bayer Schering Pharma, and Pfizer; DA has received fees for serving as co-investigator and speaker for Actelion Pharmaceuticals Ltd, Bayer, and AOP Orphan Pharmaceuticals; PP has received fees for serving as co-investigator and speaker for Actelion Pharmaceuticals Ltd, Bayer, and AOP Orphan Pharmaceuticals; AL has received honoraria and travel reimbursements from Actelion Pharmaceuticals Ltd; JJ, JP, TZ, RV, JM, MA, PB, and LD declare no competing interests.

## Authors’ contributions

PJ and AL participated in the design and co-ordination of the study, performed the statistical analysis and helped draft the manuscript. JJ, PB and LD participated in the design of the study and performed the statistical analysis. HA-H, JP, DA, TZ, RV, PP, JM and MA participated in the design and co-ordination of the study and helped draft the manuscript. All authors read and approved the final manuscript.

## Pre-publication history

The pre-publication history for this paper can be accessed here:

http://www.biomedcentral.com/1471-2466/14/45/prepub
